# Evaluation of Retinal Nerve Fiber Layer and Macular Thickness Pre- and Post-Chemotherapy With Carboplatin and Paclitaxel in Patients With Endometrial and Ovarian Cancer

**DOI:** 10.7759/cureus.43943

**Published:** 2023-08-22

**Authors:** Ju Juen Chin, Wan-Hazabbah Wan Hitam, Mei Fong Chong, Saw Joo Lee, Jing Mun Yew, Qi Zhe Ngoo

**Affiliations:** 1 Ophthalmology and Visual Sciences, School of Medical Sciences, Universiti Sains Malaysia, Kubang Kerian, MYS; 2 Ophthalmology, Hospital Universiti Sains Malaysia, Kubang Kerian, MYS; 3 Ophthalmology, Hospital Raja Permaisuri Bainun, Ipoh, MYS; 4 Gynae-Oncology, Hospital Raja Permaisuri Bainun, Ipoh, MYS; 5 Biostatistics and Research Methodology, School of Medical Sciences, Universiti Sains Malaysia, Kubang Kerian, MYS

**Keywords:** retinal nerve fiber layer, paclitaxel, ocular toxicity, macular thickness, carboplatin

## Abstract

Background

Carboplatin and paclitaxel are two standard chemotherapeutic agents known to cause neurotoxicity. In this study, we aim to evaluate the toxicity of these agents by measuring the peripapillary retinal nerve fiber layer (RNFL) and macular thickness in patients with endometrial and ovarian cancers who are receiving them.

Methods

A one-year prospective cohort study involving 28 patients who were treated intravenously with carboplatin (200-400 mg/m^2^) and paclitaxel (175 mg/m^2^) three-weekly for six cycles was conducted. RNFL and macula thickness were measured using optical coherence tomography (OCT) before the commencement of chemotherapy, after the third cycle, and one month after the sixth cycle. The main outcome measurements were the average RNFL thickness and central subfield thickness of the macula.

Results

The mean age of the 28 participants was 54.68 years old (standard deviation [SD] 9.03). Eleven had endometrial cancer, while 17 had ovarian cancer. The mean of the average RNFL thickness during baseline pre-chemotherapy was 96.43 µm (SD 11.39). One month after cessation of treatment, the mean RNFL thickness increased to 101.57 µm (SD 13.54). Statistical analysis showed a significant increment in the mean RNFL thickness (p* *≤ 0.001), from baseline to after three cycles, and baseline to one month after six cycles of chemotherapy, except the nasal quadrant. The increment of all macular quadrants was statistically significant (p < 0.05) except for central subfield thickness.

Conclusion

Systemic administration of carboplatin and paclitaxel affected both the peripapillary RNFL and macula thickness. This represents early evidence of subacute subclinical retinal toxicity. OCT can be used as a screening tool to assess peri-chemotherapeutic retinal alterations.

## Introduction

Carboplatin and paclitaxel (CP) are first-line chemotherapy drugs used worldwide to treat endometrial and ovarian cancers [1,2]. The current standard of care in advanced ovarian cancer is carboplatin (area under curve 5/6, 200-400 mg/m^2^) and paclitaxel (175 mg/m^2^) three-weekly for six cycles. This is a grade A recommendation as per the Royal College of Obstetrics and Gynecologists document [2]. For uterine cancers, the optimal chemotherapy schedule of CP is derived from similar responses of endometrial cancer to epithelial ovarian cancer and is graded D [1].

Neurotoxicity represents the major dose-limiting toxicity of CP [3-5]. Optic neuropathy and maculopathy are considered forms of neurotoxicity. Early optic nerve damage is reflected by changes in the peripapillary retinal nerve fiber layer (RNFL). Since these chemotherapeutic agents are recommended as first-line treatments in combating endometrial and ovarian cancers, it is imperative to quantify the ocular neurotoxicity effects, especially in cumulative dose regimens. To date, no published reports are available on the evaluation of the ocular changes due to toxicity during the course of chemotherapy in patients receiving CP combination.

A regular ophthalmic examination which includes visual acuity, slit-lamp biomicroscopy, and photography has been used to detect ocular toxicity during clinical practice. However, these assessments may not be useful in detecting subclinical CP-induced optic neuropathy or retinopathy. Currently, newer imaging equipment such as optical coherence tomography (OCT) can reveal changes in the RNFL and macular thickness before visual defects surface [6].

The purpose of this study is to evaluate the changes in RNFL and macula thickness in endometrial and ovarian cancer patients receiving CP at baseline before the commencement of chemotherapy, after three cycles, and one month after six cycles.

## Materials and methods

This is a prospective cohort study conducted in the ophthalmology clinic of a tertiary care center from July 2019 to July 2020. This was done in collaboration with the gynae-oncology team of the same center. This study adheres to the tenets of the Declaration of Helsinki and was approved by the local ethical boards of our university (USM/JEPeM/19010005) and the Medical Research Ethics Committee (MREC) of Malaysia (KKM/NIHSEC/P19-353(12) and NMRR-18-3438-44251 (IIR)).

All participants had given their written informed consent before their inclusion in the study. A total of 28 subjects who were newly diagnosed cases of endometrial or ovarian cancer were selected. The diagnosis was confirmed by the gynae-oncology team based on histopathological examination. These patients were planned for the commencement of chemotherapy with CP.

Patients with underlying ocular diseases such as optic nerve and macula diseases, past history of ocular surgery or trauma, current and past history of intake of other neurotoxic drugs, brain metastasis, and concurrent nervous system disorders (such as Parkinson’s disease, Alzheimer’s disease, stroke, and bipolar disorder) were excluded.

The nature of the study was explained to the subjects who fulfilled the selection criteria, and written consent was obtained. All patients underwent a comprehensive ophthalmological examination that included best-corrected visual acuity (BCVA), slit-lamp examination, applanation tonometry, dilated fundus examination, and OCT. They underwent these examinations at three different times, which were at baseline (pre-chemotherapy), after three cycles, and after six cycles of chemotherapy (one-month post cessation of chemotherapy). Patients who had an abnormality in the initial OCT were excluded from this study. The same OCT machine was used for measurement throughout the entire study.

The peripapillary RNFL thickness and macular thickness were performed by a single qualified and trained personnel using Stratus OCT (Carl Zeiss Meditec Inc., Dublin, CA). Good quality scans comprising a signal strength of more than five were used, with proper centering and focused images. Only the findings of the right eye of each patient were used.

All statistical analyses were calculated using the Statistical Package for Social Sciences (SPSS) version 26 (IBM Corp., Armonk, NY). Descriptive statistics were conducted to describe the demographic profile. For repeated measures, ANOVA was used to compare the changes in RNFL and macula thickness at pre-chemo, after three cycles, and one month after six cycles. The quantitative data was expressed as mean (standard deviation) since it showed a normal distribution. A p-value < 0.05 was considered statistically significant.

## Results

The mean age of the 28 participants was 54.68 years (standard deviation [SD] 9.03). Eleven out of 28 patients (39.3%) were diagnosed with endometrial cancer, while 17 out of 28 patients (60.7%) had ovarian cancer. The majority of the subjects were Malay (50%, n = 14). Distribution of age, race, presence of comorbidities, and type of cancer are shown in Table 1.

**Table 1:** Demographic variables between respondents (n = 28)

	Mean (SD)	Frequency (%)
Age	54.68 (9.03)	
Race		
Malay		14 (50.0)
Chinese		11 (39.3)
Indian		3 (10.7)
Presence of medical problem		
Nil		11 (39.3)
Present		17 (60.7)
Diabetes mellitus		
Nil		21 (75.0)
Present		7 (25.0)
Hypertension		
Nil		18 (64.3)
Present		10 (35.7)
Dyslipidemia		
Nil		19 (7.9)
Present		9 (32.1)
Type of cancer		
Endometrial		11 (39.3)
Ovarian		17 (60.7)

Each patient received intravenous carboplatin (area under curve 5/6, 200-400 mg/m^2^) and paclitaxel (175 mg/m^2^) consecutively, over a four-hour period, every three-weekly for a maximum of six cycles of treatment. The ophthalmological examinations that included BCVA, slit-lamp examination, applanation tonometry, and dilated fundus examination were normal during each visit in all of our patients. There was no significant difference (p = 0.367) in the intraocular pressure (IOP) before, during, or after chemotherapy (15.00, 14.86, and 14.71 mmHg, respectively).

The mean of the average RNFL thickness during baseline pre-chemotherapy was 96.43 µm (SD 11.39). One month after cessation of treatment, the mean RNFL thickness increased to 101.57 µm (SD 13.54). The difference was statistically significant in all quadrants except nasal RNFL (p = 0.073). This is shown in Table 2.

**Table 2:** Comparison of mean RNFL thickness of the right eye between times among patients with endometrial and ovarian cancer (n = 28) ^a^ Tested by repeated measure ANOVA. * Level of significance at p < 0.05. RNFL: Retinal nerve fiber layer; ANOVA: Analysis of variance.

Parameters (μm)	Pre-chemotherapy	After 3 cycles	After 6 cycles	F statistics (df)	p-values^a^
	Mean (SD)	Mean (SD)	Mean (SD)		
Average	96.43 (11.39)	99.96 (12.68)	101.57 (13.54)	10.0 (2, 54)	<0.001*
Superior	119.29 (18.74)	120.82 (22.16)	124.79 (23.75)	5.23 (1.7, 45.9)	0.012*
Nasal	68.82 (9.39)	71.79 (9.67)	73.36 (11.72)	2.97 (1.6, 42.6)	0.073
Inferior	124.64 (26.06)	128.79 (24.19)	130.39 (25.61)	4.65 (2, 54)	0.014*
Temporal	73.07 (13.88)	78.36 (13.63)	78.04 (12.60)	12.22 (1.5, 41.5)	<0.001*

Statistical analysis showed a significant increment between pre- and post-chemotherapy in the mean RNFL thickness (p ≤ 0.001) from baseline to after three cycles and from baseline to one month after six cycles of chemotherapy. This is shown in Table 3.

**Table 3:** Mean difference of measurements in the average RNFL thickness of the right eye among patients with endometrial and ovarian cancer (n = 28) ^b^ Repeated measure after Bonferroni correction. * Level of significance at p < 0.05. RNFL: Retinal nerve fiber layer.

Comparisons	Average RNFL thickness	
	Mean difference (95% CI)	p-values^b^
Baseline to 3 cycles	3.54 (0.34, 6.74)	0.027*
Baseline to 6 cycles	5.14 (1.73, 8.55)	0.002*
3-6 cycles	1.61 (-0.67, 3.88)	0.248

**Figure 1:**
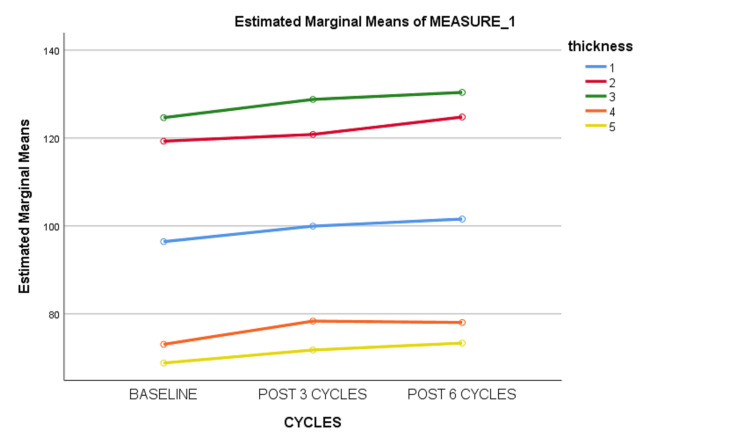
Mean of RNFL thickness with time 1: Average; 2: Superior; 3: Inferior; 4: Temporal; 5: Nasal. RNFL: Retinal nerve fiber layer.

Repeated measure ANOVA showed that there was a significant increment in the mean of RNFL thickness with chemotherapy cycles as shown in Figure 1.

The mean of the average central subfield thickness of the macula during baseline pre-chemotherapy was 230.93 µm (SD 23.40). This increased to 243.36 µm (SD 22.31) one-month post-chemotherapy. The difference was not statistically significant (p = 0.321). However, the difference was significant for all other quadrants (p < 0.05). This is shown in Table 4.

**Table 4:** Comparison of the mean macular thickness of the right eye between times among patients with endometrial and ovarian cancer (n = 28) ^a^ Tested by repeated measure ANOVA. * Level of significance at p < 0.05. ANOVA: Analysis of variance.

Parameters (μm)	Pre-chemotherapy	After 3 cycles	After 6 cycles	F statistics (df)	p-values^a^
	Mean (SD)	Mean (SD)	Mean (SD)		
Central subfield	230.93 (23.40)	233.25 (22.63)	243.36 (22.31)	1.09 (1.3, 34.1)	0.321
Superior inner	308.36 (16.56)	314.43 (16.47)	316.75 (17.51)	14.02 (1.7, 46.1)	<0.001*
Outer	274.68 (16.11)	284.82 (13.08)	285.68 (15.81)	26.81 (1.7, 46.6)	<0.001*
Temporal inner	297.00 (16.10)	303.25 (17.12)	303.11 (16.94)	11.41 (2, 54)	<0.001*
Outer	254.93 (13.01)	264.54 (13.62)	261.54 (13.17)	12.48 (2, 54)	<0.001*
Nasal inner	311.18 (19.47)	317.50 (19.38)	319.25 (19.02)	8.59 (2, 54)	0.001*
Outer	294.50 (15.53)	299.86 (22.37)	305.50 (16.69)	4.12 (1.3, 35.5)	0.040*
Inferior inner	306.18 (16.78)	312.25 (17.63)	313.07 (16.99)	18.03 (2, 54)	<0.001*
Outer	264.61 (14.87)	269.86 (15.23)	274.14 (14.99)	4.499 (2, 54)	0.016*

Statistical analysis showed that there was no significant increment between pre- and post-chemotherapy in the mean macular central subfield thickness. This is shown in Table 5.

**Table 5:** Mean difference of measurements in the macula central subfield thickness of the right eye among patients with endometrial and ovarian cancer (n = 28) ^b^ Repeated measure after Bonferroni correction. * Level of significance at p < 0.05.

Comparisons	Average macular central subfield thickness	
	Mean difference (95% CI)	p-values^b^
Baseline to 3 cycles	2.32 (-0.66, 5.31)	0.172
Baseline to 6 cycles	3.43 (-3.93, 10.78)	0.733
3-6 cycles	1.11 (-5.76, 7.97)	1.000

However, the increment in the thickness of all other quadrants was statistically significant from baseline to after three cycles and baseline to one-month post six cycles of chemotherapy as shown in Table 6 (p < 0.05).

**Table 6:** Mean difference of measurements in the macula thickness of the right eye among patients with endometrial and ovarian cancer (n = 28) ^b^ Repeated measure after Bonferroni correction. * Level of significance at p < 0.05.

Measurements	Comparisons					
	Baseline to 3 cycles		Baseline to 6 cycles		3-6 cycles	
	Mean difference (95% CI)	p-values^b^	Mean difference (95% CI)	p-values^b^	Mean difference (95% CI)	p-values^b^
Superior						
Inner	6.07 (1.79, 10.36)	0.004*	8.39 (3.46, 13.33)	0.001*	2.32 (-0.79, 5.44)	0.204
Outer	10.14 (5.89, 14.39)	<0.001*	11.00 (5.93, 16.07)	<0.001*	0.86 (-2.43, 4.15)	1.000
Temporal						
Inner	6.25 (1.85, 10.65)	0.004*	6.11 (2.12, 10.10)	0.002*	0.14 (-2.75, 3.03)	1.000
Outer	9.61 (3.97, 15.24)	0.001*	6.61 (1.90, 11.31)	0.004*	3.00 (-1.66, 7.66)	0.337
Nasal						
Inner	6.32 (0.61, 12.03)	0.026*	8.07 (2.21, 13.94)	0.005*	1.75 (-2.13, 5.63)	0.778
Outer	5.36 (-6.25, 16.96)	0.747	11.00 (6.10, 15.91)	<0.001*	5.64 (-5.70, 16.98)	0.645
Inferior						
Inner	6.07 (2.49, 9.65)	0.001*	6.89 (3.50, 10.29)	<0.001*	0.82 (-1.70, 3.35)	1.000
Outer	5.25 (-2.09, 12.59)	0.237	9.54 (1.14, 17.93)	0.022*	4.29 (-4.31, 12.88)	0.641

**Figure 2:**
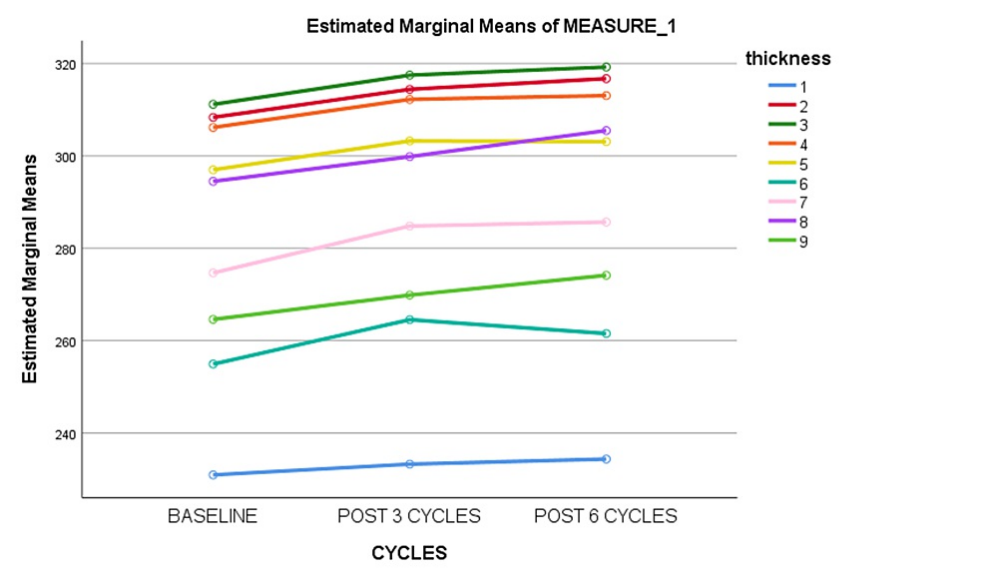
Mean of macular thickness with time 1: Macular central subfield thickness; 2: Inner superior; 3: Inner nasal; 4: Inner inferior; 5: Inner temporal; 6: Outer temporal; 7: Outer superior; 8: Outer nasal; 9: Outer inferior.

Repeated measure ANOVA showed that there was no significant increment in the mean of macular central subfield thickness with chemotherapy cycles. However, it showed that there was a significant increment in all other quadrants of mean macular thickness with chemotherapy cycles (Figure 2).

## Discussion

In our study, the mean age of the 28 participants was 54.68 years (SD 9.03). This is in accordance with the data obtained from the latest Malaysia National Cancer Registry Report 2012-2016 published in 2017 [7]. The majority of the patients were Malay (50%, n = 14), followed by Chinese (39.3%, n = 11) and Indian (10.7%, n = 3). Most of them had underlying medical problems such as diabetes mellitus, hypertension, and dyslipidemia (60.7%, n = 17).

As the optic nerve fibers are affected by elevated IOP, we included IOP measurements at each examination time. We found no differences in IOP at baseline, during, or after completion of chemotherapy (p = 0.367). All our patients also had no ocular complaints during each visit, and their BCVA was stable throughout the course of chemotherapy.

The human eye is small and has a rich vascular supply. This makes it particularly vulnerable to systemically administered drugs. The major hurdle for systemic drugs to reach the eye is the blood-ocular barriers, namely the blood-aqueous barrier and blood-retina barrier. Optic neuropathy and macular degeneration can occur following systemic drug administration.

In this pilot study, we found evidence that supports the previous knowledge of ocular toxicity in patients treated with CP. OCT measurements of peripapillary RNFL thickness and macular thickness (all except the foveal center) both showed statistically significant increments over time through the course of chemotherapy. The RNFL is made up of axons of retinal ganglion cells. They carry electrical impulses from the retina to the optic nerve and optic chiasm. RNFL thickness is a morphological measurement that can be affected in the early stages of many optic nerve conditions such as glaucoma and optic neuropathy. A decrease in thickness implies a loss of axons of ganglion cells. An increase in thickness is found in cases of inflammation or toxicity. Early changes can be detected using OCT before any visual field defects appear [6]. The OCT machine is a widely used diagnostic tool, and it is commonly available in ophthalmology clinics in this era. It provides cross-sectional and three-dimensional images of layered retinal structures. OCT provides a quantitative tool for the comparison of RNFL and macula in the human retina to a database of known normal subjects. It does this by measuring the echo time delay of backscattered light signals via an interferometer. Using near-infrared light, the OCT provides quantitative and reproducible measurements of RNFL and macular thickness parameters [8]. For the past few years, spectral domain OCT was introduced, taking over the popularity of time-domain OCT usage in clinical practice. This is attributed to its higher-resolution images, greater reproducibility, and better scan acquisition speed. Faster speed captures better images with clear delineation of retinal structures and reduces eye motion artifacts [9]. Cirrus high definition (HD) OCT (Carl Zeiss Meditec, Jena, Germany) is a Fourier domain OCT (FDOCT) with an axial resolution of 5 µm and a scan velocity of 27,000 axial scans per second.

The increment in the peripapillary RNFL thickness was statistically significant from baseline to three cycles and baseline to one-month post chemotherapy with CP. An increment in the RNFL thickness, while the patient is receiving CP chemotherapy, is indicative of subclinical retinal toxicity. Similar increments in RNFL thickness were found with other ocular drug toxicities, mainly ethambutol and hydroxychloroquine [10-12]. They postulated that this might be explained by ethambutol causing mitochondrial dysfunction, which leads to reduced axonal transport, particularly affecting small caliber parvo-cellular retinal ganglion cell axons of the optic nerve and papillomacular bundle. This was based on an in vitro Leber’s hereditary optic neuropathy (LHON)-mimicking mice model study report [13-15]. The mechanism of ocular neurotoxicity with CP remains unknown. Previous studies suggested retinal vascular dysregulation or optic nerve ischemia to be the possible mechanism. Electrophysiological changes in patients with reversible scotoma were comparable to those changes observed in ischemic neuropathies, suggesting that the optic nerve may be the main target site of CP [16,17].

Bakbak et al. assessed CP-associated toxicities in the optic nerve by measuring the RNFL thickness and visual field changes in patients with lung cancer [18]. They found statistically significant decrement in RNFL thickness and visual field changes recorded by OCT and Humphrey visual field analyzer (HFA), respectively. The measurement of RNFL was done at baseline and three months after cessation of chemotherapy. In contrast to their results, we found an increment in the RNFL thickness instead. This could possibly be explained by the timing at which the RNFL thickness was measured. Ours was measured at different times throughout the chemotherapy cycles and reflected the thickness during and one month after cessation. This occurred when blood levels of CP were highest; therefore, acute ongoing subclinical ocular toxicity was reflected by an increase in the RFNL thickness. This might be due to edema or inflammation of the RNFL; however, in their study, their last measurement was taken three months post cessation of CP when edema would have subsided and the loss of ganglion cell axons due to CP toxicity was reflected by the decrement in RNFL thickness. Another similar study by Dulz et al. investigating the ocular toxicity of cisplatin-based chemotherapy in patients with germ cell cancer showed similar decrements in RNFL thickness, which correlated with cumulative cisplatin doses [19]. This shows that alterations in RNFL thickness reflect the underlying subclinical retinal toxicity of CP.

In our study, there was also a statistically significant increment in macular thickness throughout the chemotherapy with CP. This supports the existing knowledge on paclitaxel causing angiographically silent macular edema [20]. The mechanism of this edema is not clearly understood. One theory suggests that the permeability of retinal vessels due to the breakdown of the blood-ocular barrier is so minute that larger fluorescein molecules are unable to pass through [21]. Another theory explains that it is due to the toxicity of Muller cells, which leads to disturbances in the osmotic gradient within the neurosensory retina and results in intracellular fluid accumulation [22].

Taxanes are well known to cause fluid retention that worsens with cumulative doses [23]. This can manifest as edema, weight gain, and third-space fluid retention (pericardial, pleural, and ascites). In cases of cystoid macular edema occurring without the presence of systemic fluid retention, it was suggested that this occurred as a result of cellular toxicity derived from the suppression of intracellular microtubule reorganization by taxanes [24]. In our study, the macular edema is subclinical, which is reflected by an increase in the overall thickness but without any changes in the normal layered structure of the macula. The increment in the central subfield thickness was not statistically significant probably due to the fact that the fovea is an avascular zone, so systemically administered chemotherapy drugs will have less effect on this region.

Platinum-based chemotherapy is used in a wide spectrum of solid malignancies other than uterine and ovarian cancer. They are also used in lung cancer, germ cell tumors, bladder cancer, and head and neck cancers. All the main clinical guidelines (American Society of Clinical Oncology [ASCO5] and European Society of Medical Oncology [ESMO]) recommend first-line cisplatin-based chemotherapy as the treatment of choice in both advanced cancers and neoadjuvant chemotherapy [25].

Cisplatin is a cytotoxic heavy-metal compound. It induces apoptosis of tumor cells by substituting hydrogen atoms with alkyl groups [26]. Drugs that can be combined with platinum include third-generation cytotoxic drugs such as docetaxel, paclitaxel, gemcitabine, irinotecan, and pemetrexed. Neurotoxicity is the major dose-limiting toxicity of cisplatin. Its other well-known complications are nephrotoxicity and peripheral neuropathy [27].

Carboplatin has a similar mechanism of action with a more favorable toxicity profile [28]. It is superior to cisplatin due to reduced toxicity and deliverability [1]. Visual impairment has been considered a rare and infrequent form of neurotoxicity. Long-term intravenous carboplatin therapy causes various ocular complications. Severe orbital inflammation, proptosis, loss of eye movements, loss of vision, optic neuropathy, maculopathy, sore eyes, and chorioretinitis to optic neuritis have been described [29-31]. Previous studies showed reversible visual disturbances after drug cessation. However, in a case report and literature review by Li et al., they described a case of irreversible blindness caused by CP therapy and concluded after a review of literature that CP-induced ocular toxicity is usually irreversible [5]. Another recent study by Dulz et al. also found that retinal damage was irreversible as there was no tendency toward an improvement of average RNFL thickness after cessation of cisplatin-based chemotherapy [19]. They found evidence for subclinical dose-dependent toxic retinal alterations in patients treated with cisplatin-based chemotherapy for germ cell cancer.

Paclitaxel belongs to the taxane class of chemotherapy drugs. It is a mitotic inhibitor that stabilizes microtubules and interferes with their usual breakdown during cell division [18]. Other than its use in uterine and ovarian cancer, it is also widely used in lung cancer. The neurotoxicity of paclitaxel is widely known. Visual impairment and transient scintillating scotoma have been reported. Reversible scotoma with visual evoked potential abnormalities similar to that of demyelinating optic neuropathy was reported by Capri et al. [32]. Photopsia has been reported to occur during the last half an hour of intravenous infusion of paclitaxel. This resolves totally within three hours and usually occurs with higher doses of 250 mg/m^2^ or more [33]. It rarely occurs at 175 mg/m^2^ doses used in the treatment of uterine and ovarian cancers.

As both carboplatin and paclitaxel are well known for their cumulative dose toxicity profiles, they may have a synergistic effect to potentiate optic nerve and macula damage when used together. Till today, it is not known whether the combination of these chemotherapeutic drugs will potentiate optic nerve damage as compared to administering a single drug [5,18].

We noticed some limitations in our study. It lacks a long-term measurement and functional impact assessment. This study only spans from pre-chemotherapy to one-month post cessation of chemotherapy with CP. In future studies, the RNFL thickness should be evaluated further for a longer duration to determine the severity and clinical impact of retinal alterations related to this chemotherapy regimen. Also, the co-administration of CP makes it impossible to point out which of these agents is the culprit in contributing to this subclinical retinal damage. Moreover, our study only evaluated the morphological changes and not the functional changes induced by CP. We did not evaluate the visual electrophysiology tests in this study because the tests are not available in one of our study centers. Visual electrophysiology tests such as visual evoked potential (VEP) and electroretinogram (ERG) are used to evaluate the function of the visual pathway from the retina to the occipital cortex. These tests may aid in the detection of subclinical retinal toxicity. We suggest the tests be carried out in future studies so that a complete structural and functional assessment can be obtained.

From a clinical point of view, these subclinical changes should be informed to every patient prior to chemotherapy with CP. Patients should understand that these effects might be clinically relevant in the presence of other co-existing retinal neurodegenerative disorders such as optic neuropathy or glaucoma. Complete ophthalmological assessment including OCT measurements could be considered at baseline and at regular intervals during chemotherapy. Though rare, gynae-oncologist should be alert to the possibility of ophthalmic adverse events occurring due to CP chemotherapy in this vulnerable group of gynae cancer patients.

## Conclusions

In conclusion, this paper describes the first study evaluating RNFL thickness and macula thickness over time in patients who received CP chemotherapy for endometrial and ovarian cancer. Our study showed that systemic administration of these agents affected both the peripapillary RNFL and macula thickness and hence represents early evidence of subacute subclinical retinal toxicity. Hence, OCT can be used as a screening tool to assess peri-chemotherapeutic retinal alterations. Further studies including assessment of functional visual changes, electrophysiology, and long-term follow-up would help to provide better evidence of acute and chronic ocular toxicity caused by these agents.
